# Does ocular treatment of uveal melanoma influence survival?

**DOI:** 10.1038/sj.bjc.6605765

**Published:** 2010-07-27

**Authors:** B Damato

**Affiliations:** 1 Ocular Oncology Service, Royal Liverpool University Hospital, Prescot St, Liverpool L7 8XP, UK

**Keywords:** surgery, research, clinical trials, oncology, uveal melanoma

## Abstract

Treatment of uveal (intraocular) melanoma is aimed at prolonging life, if possible conserving the eye and useful vision. About 50% of patients develop fatal metastatic disease despite successful eradication of the primary intraocular tumour. The effect of ocular treatment on survival is unknown, because the same survival data from case series can be interpreted in different ways. Treatment is therefore based on intuition and varies greatly between centres. Randomised trials of treatment *vs* non-treatment of asymptomatic tumours are desirable but would be controversial, difficult, expensive and possibly inconclusive. Strategies for coping with uncertainty are needed to avoid unethical care.

It is widely believed that the primary reason for treating uveal melanomas is to prevent metastatic spread of tumour from the eye to other parts of the body. Despite successful eradication of the ocular tumour, however, about 50% of all patients develop metastatic disease, which is almost invariably fatal (Kujala *et al*, 2003). In this article, I discuss different ideas as so how treatment of uveal melanomas influences survival. It is hoped that some of the lessons learnt from the experience with these tumours will be relevant to other fields of surgical oncology.

## Uveal melanoma

### Epidemiology

Uveal melanomas have an incidence of approximately 2-8 per million per year in Caucasians. An ophthalmologist in such communities will see a new patient with this disease only rarely. Presentation is at a median age of 60 years. Men and women are affected the in equal numbers. Disease is most common in individuals with a fair complexion and a blue or grey iris colour. The role of sunlight is uncertain.

More than 90% of uveal melanomas involve the choroid, causing detachment of the overlying retina. The remainder are confined to the iris and ciliary body and tend to cause cataract and glaucoma. Most patients therefore present with blurred vision or visual field loss. Advanced melanomas can make the eye inflamed and painful. Extraocular extension into the orbit can occur at any stage.

### Detection of uveal melanomas

In UK, about 30% of patients are asymptomatic at the time of diagnosis, their tumour being detected on routine examination; conversely, about 20% of symptomatic patients report that their tumour was initially missed (Damato, 2001). Studies in other countries such as Finland and the USA have reported similar results (Eskelin and Kivelä, 2002; Bove and Char, 2004). In addition, many patients experience delays in the referral process, because of administrative shortcomings and diagnostic errors.

## Ocular treatment of uveal melanoma

Ocular treatment of uveal melanoma is termed ‘radical’ if it consists of enucleation (i.e., ocular amputation) and ‘conservative’ if conservation of useful vision and the eye is attempted (Damato, 2006).

In most centres, the first choice of conservative treatment is brachytherapy, delivered using plaque-shaped applicators containing isotopes such as iodine-125 or ruthenium-106, which emit *γ-* and *β*-irradiation respectively. The plaque is sutured to the sclera overlying the tumour and removed days later once the required dose of at least 80 Gy has been delivered to the tumour. Facilities for proton beam radiotherapy are becoming available in a growing number of centres, some administering this treatment to all patients and others reserving it for patients whose tumour is considered unsuitable for brachytherapy. Stereotactic radiotherapy is becoming more widespread in centres that are not able to administer proton beam treatment.

After radiotherapy, many uveal melanomas become exudative, causing visual loss from macular oedema and retinal detachment, which if severe results in iris neovascularisation and painful glaucoma (i.e., ‘toxic tumour syndrome’). These complications are treated with intraocular injections of steroids or anti-angiogenic agents or by administering phototherapy to the irradiated tumour or removing it surgically.

*En bloc* tumour resection is widely performed for small iris and ciliary body melanomas (i.e., iridectomy, iridocyclectomy, cyclectomy); however, only a few surgeons excise large ciliary body and choroidal melanomas (i.e., choroidectomy, cyclochoroidectomy). This is because such surgery is technically difficult, also requiring profound systemic hypotensive anaesthesia.

Choroidal melanomas can also be removed by ‘endoresection’, which involves piecemeal tumour resection using a vitreous cutter that is passed through the retina, which is subsequently ‘welded’ into place with laser treatment. This operation is highly controversial without neoadjuvant radiotherapy, because of concerns about disseminating malignant cells around the eye and systemically.

Small, pigmented choroidal melanomas can be treated by transpupillary thermotherapy, whereby an infrared laser beam is used to induce hyperthermia for about 60 s. Photodynamic therapy is still an investigational procedure but seems effective with some small, amelanotic tumours.

Increasingly, multi-modality treatment is administered, for example, performing tumour resection with adjunctive or neoadjuvant radiotherapy or combining radiotherapy with phototherapy.

Large naevi can be clinically indistinguishable from small melanomas. It is accepted practice for such ‘suspicious naevi’ or ‘melanocytic tumours of indeterminate malignancy’ to be left untreated until growth has been documented by sequential ophthalmoscopy or ultrasonography.

**Figure 1:**
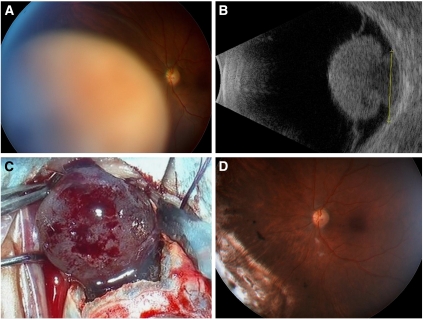
(**A**) A 44-year-old man was referred with an inferonasal choroidal melanoma in the left eye, which had a visual acuity of 6/7.5. (**B**) On ultrasonography, the tumour had a collar-stud shape and measured 15.3 mm in basal diameter, with a thickness of 11.2 mm. (**C**) The patient underwent transscleral local resection under hypotensive anaesthesia with adjunctive ruthenium plaque brachytherapy. (**D**) At 6 months postoperatively, the visual acuity was 6/9.5. The melanoma was of spindle-cell type with no chromosome 3 loss shown by multiplex ligand-dependent probe amplification. These findings indicate a minimal risk of metastatic disease and therefore a near-normal life expectancy.

Outcomes such as ocular conservation, visual preservation and local tumour control depend greatly on the tumour size, proximity to optic disc or fovea, extent of ciliary body or angle involvement, presence or absence of extraocular spread and ocular morbidity such as retinal detachment, glaucoma and cataract (Figure 1). In specialist centres, ocular preservation is attempted in about 60–70% of patients and is successful in about 90% of these cases (Damato and Lecuona, 2004). Actuarial rates of local tumour recurrence range from less than 5% after plaque or proton beam radiotherapy to approximately 10–15% after phototherapy or transscleral choroidectomy especially. Some degree of useful vision is preserved in most patients, if the tumour does not extend close to disc or fovea.

Ocular treatment varies greatly between centres, not only according to the expertise and equipment available in each centre but also according to the rationale of treatment, which depends on how treatment is believed to influence survival. For these reasons, few randomised, controlled trials have been conducted to compare one treatment with another so that most evidence is based on case series.

## Metastatic disease

Metastatic spread occurs haematogenously. About 90% of patients with metastatic disease show hepatic involvement, other sites including lung, skin, bone and brain. Death usually occurs within a year of the onset of systemic symptoms (Eskelin *et al*, 2003). Treatment by systemic or intra-hepatic chemotherapy or partial hepatectomy only rarely prolongs life (Augsburger *et al*, 2009).

There is controversy about whether to screen for metastatic disease and if so in whom and for how long. When screening is undertaken, there is great variation in the methods used, with some relying on blood tests and others performing imaging consisting of ultrasonography, magnetic resonance imaging or positron-emission tomography.

Because many patients already have micrometastases by the time their ocular tumour treatment is detected and treated, some groups have investigated various forms of systemic adjuvant therapy in high-risk patients. No definite survival benefit has been demonstrated although one study showed a non-significant trend (Desjardins *et al*, 1998; Voelter *et al*, 2008).

## Estimating the survival prognosis

As with other cancers, estimation of the survival probability allows patients with a good prognosis to be reassured about their life expectancy while targeting special measures at high-risk patients. Prognostication also allows patients with low risk of metastasis to be excluded from studies evaluating the impact of ocular or systemic treatment on survival, thereby enhancing the chances of detecting any statistical significance in outcome.

The most important factors predicting metastatic disease are: (1) basal tumour diameter; (2) ciliary body involvement; (3) transscleral extension; (4) epithelioid melanoma cytomorphology; (5) high mitotic rate; (6) extravascular matrix patterns such as closed loops; (7) microvascular density; (8) chromosome 3 deletion, chromosome 8q gain and lack of chromosome 6p gain and (9) a class 2 gene expression (Prescher *et al*, 1996; White *et al*, 1998; Onken *et al*, 2004; Damato *et al*, 2007; Damato and Coupland, 2009).

Survival studies have largely relied on clinical features for prognostication, particularly the largest basal tumour diameter. However, tumours having the same dimensions show significant variation in survival according to their histological and genetic features (Damato and Coupland, 2009). The author and associates have therefore developed online neural networks for generating personalised survival curves using clinical, histological and genetic predictors, also taking age and sex into account (www.ocularmelanomaonline.com; Damato *et al*, 2008). Such multivariate analysis has enhanced the reliability of prognostication so that it is relevant to individual patients.

Improvements in surgical, laboratory and statistical methods have increased the scope of prognostication so that genetic tumour typing is now being performed in a growing number of centres, not only with tumours treated by enucleation or local resection but also in patients undergoing radiotherapy or phototherapy. Such patients may have transscleral or transretinal biopsy immediately before or after treatment.

In the absence of a good treatment for metastatic disease, opinions vary as to how much patients should be told about their prognosis and hence whether tumour biopsy for prognostication is justifiable.

## Effect of ocular therapy on survival

### Hypothesis 1: timely enucleation prevents metastatic death

**Figure 2:**
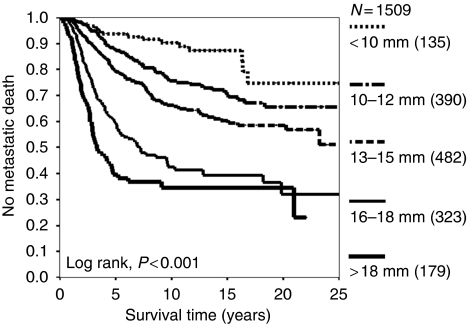
Kaplan–Meier survival curves showing rates of metastatic death according to basal tumour diameter after treatment of choroidal melanoma. Patients were included only if residing in mainland UK and if histological tumour type was known (from Damato and Coupland, 2009).

For more than a century, uveal melanomas were treated radically, by urgent enucleation, so as to maximise any chances of survival. It was generally accepted that ocular treatment successfully prevented metastatic spread in many patients, particularly if the treatment was performed early, when the tumour was still small. Strong correlations between tumour size and metastatic death supported this view (Figure 2).

### Hypothesis 2: conservative forms of ocular treatment are dangerous

Manschot, a vociferous Dutch pathologist, condemned radiotherapy as unsafe and strongly recommended enucleation for all patients (Manschot and van Strik, 1987). His advice was based on the finding that viable tumour cells can be seen in most irradiated melanomas. However, several non-randomised studies showed no statistical difference between conservative and radical forms of therapy. Conservative forms of therapy were generally considered ethical if the patient was prepared to take a risk to avoid mutilating surgery or visual handicap.

### Hypothesis 3: ocular treatment accelerates metastatic death

In 1978, Zimmerman, an eminent American pathologist, published an influential article suggesting that enucleation (and by implication, other treatments) accelerated metastatic death by physically disseminating tumour cells from the eye into the general circulation (Zimmerman *et al*, 1978). This hypothesis was based largely on the observation that the mortality rate peaks in the second post-operative year. Concerns about iatrogenic tumour dissemination led to several preventative measures, such as pre-enucleation radiotherapy. Some abandoned treatment altogether unless the eye became painful.

### Hypothesis 4: brachytherapy and enucleation are equally effective

To resolve controversies about Zimmerman is and Manschot's hypotheses, forty centres in North America undertook large, randomised, multi-centre, collaborative ocular melanoma studies (COMS). One of these studies investigated the impact of pre-enucleation, external beam radiotherapy on survival in 1003 patients with a large uveal melanoma (Hawkins, 2004). This trial concluded that it found no survival advantage attributable to pre-enucleation radiotherapy. The implication of this result was that Zimmerman's hypothesis was wrong and that traumatic dissemination of malignant cells at the time of enucleation did not accelerate death. The most plausible explanation for the observed peak in metastatic death in the second post-operative year is the tendency for patients to present when the tumour diameter is 13 mm.

Another COMS trial involving 1317 patients compared survival after iodine plaque radiotherapy with that after enucleation, in patients with a medium-sized melanoma (Collaborative Ocular Melanoma Study Group, 2006). Recruitment took longer than expected because many patients were reluctant to lose their eye when less disfiguring treatment was possible. Eventually, this COMS trial concluded that there was no survival difference between plaque radiotherapy and enucleation. This has been widely taken to mean that plaque radiotherapy and by implication other forms of conservative therapy are as safe as enucleation.

### Hypothesis 5: delayed treatment and local treatment failure increase mortality

Straatsma *et al* (2003) compared 43 untreated patients with historical controls and reported a trend towards higher mortality in patients who were not immediately treated; however, this result may have been caused by selection bias as the patients with deferred treatment tended to be older. Gragoudas *et al* (2002) have shown local tumour recurrence after conservative treatment to be associated with higher mortality, suggesting that the recurrences shortened life, however, others countered this argument with the suggestion that recurrences were merely an indictor of increased tumour malignancy.

### Hypothesis 6: survival is determined by genetic melanoma type and not by treatment

**Figure 3:**
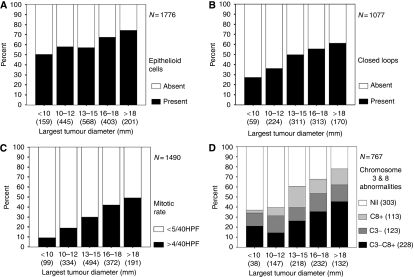
Prevalence of choroidal melanomas with (A) epithelioid cells, (B) closed connective tissue loops, (C) high mitotic rate and (D) chromosome 3 loss, according to basal tumour diameter (from Damato and Coupland, 2009). HPF, high-power fields.

In the 1990s, it was discovered that uveal melanomas tend to develop several non-random chromosomal abnormalities, particularly chromosome 3 loss (monosomy-3) and gains in 6p and 8q. In 1996, Prescher *et al* found that metastatic death occurred exclusively in patients with a monosomy-3 melanoma. Later studies reported that chromosome 3 loss and chromosome 6p gain to be mutually exclusive (Parrella *et al*, 1999). The concept arose that there are two distinct types of uveal melanoma: lethal, monosomy-3 melanomas and non-lethal, disomy-3 melanomas, the latter often showing chromosome 6p gain (Tschentscher *et al*, 2003). Estimates of tumour doubling times of metastases from uveal melanomas supported the hypothesis that lethal melanomas metastasise when they are very small, several years before the ocular tumour is detected and treated (Eskelin *et al*, 2000). These inferences cast considerable doubts on the efficacy of ocular treatment in prolonging life. The correlation between tumour size and increased mortality (Figure 3) was attributed to the higher prevalence of monosomy-3 in large tumours rather than to any beneficial therapeutic effect (Augsburger *et al*, 2007; Damato and Coupland, 2009). According to this view, uveal melanomas become large after developing monosomy-3 and not before so that correlations between size and mortality reflect rate of tumour growth.

Proponents of this hypothesis argued that whether or not the COMS conclusions were correct, they were statistically inconclusive because many patients had a non-lethal melanoma so that the required statistical power was not achieved. According to this reasoning, some studies evaluating systemic adjuvant therapy would perhaps have shown a statistically significant benefit if genetic tumour typing had been undertaken to exclude non-lethal melanomas.

### Hypothesis 7: ocular treatment prevents metastasis in some patients

In 1998, White *et al* proposed that metastatic disease occurs rarely or not at all unless monosomy-3 and chromosome 8q gain occur together (White *et al*, 1998). Subsequent multivariate analyses did not support this (Damato *et al*, 2007; Ehlers *et al*, 2008). It was therefore assumed by some that monosomy-3 was the lethal abnormality and that chromosome 8q gain merely accelerated metastatic death, possibly because of increased expression of the *C-MYC* gene. Tentative data from recent studies, however, have supported findings by White *et al* that metastatic death is rare except when chromosome 3 loss and 8q gain occur together (Damato *et al*, 2009).

It is not known how many tumours without chromosome 3 loss and 8q gain at the time of treatment would have developed these changes is left untreated. It is therefore not known how many lives have been saved by timely treatment. In other words, after studying the statistics given in Figure 2 one cannot estimate to what extent survival is influenced by treatment. The concept of ‘crescendo malignancy’ is supported by intra-tumoral genetic heterogeneity, which suggests an ongoing evolutionary process (Dopierala *et al*, 2010). Furthermore, the author recently treated a patient, whose tumour suddenly grew dramatically after several years of apparent dormancy, making the eye blind and painful so that enucleation was required (Callejo *et al*, submitted). Histology and genetic studies showed the base of the tumour to consist of low-grade, spindle-cell melanoma with only partial chromosome 3 loss whereas the apical region showed high-grade, epithelioid cells with monosomy-3 and gains in chromosome 8q. This case suggests late development of the metastatic genotype, which occurred while the patient was being observed and which might have been prevented by early treatment.

According to this hypothesis, there are three groups of uveal melanoma: (1) metastasing melanomas, which have already metastasised by the time of ocular treatment even though the metastases may not be detectable; (2) pre-metastasising melanomas, which develop metastatic capability and disseminate if treatment is delayed and (3) non metastasising melanomas, which do not metastasise, even if never treated.

### Which hypothesis is correct?

It might seem that the various hypotheses are mutually exclusive, so that if one endorses one view one must necessarily exclude all other models. If uveal melanomas behave in a diverse manner, however, with some metastasising early and others very late or not at all, then more than one hypothesis may be correct and the seemingly rival hypotheses may actually complement each other to some extent.

## Coping with uncertainty

Uncertainty about the effect of ocular treatment on survival is unsettling. On the one hand, there are concerns that many patients might be sacrificing their vision and even the eye unnecessarily in the hope of living longer; conversely, there are fears that patients with a small melanoma might be dying of metastasis because treatment is delayed until growth is documented.

### Research

In theory, there is much scope for randomised trials of treatment *vs* non-treatment of asymptomatic uveal melanoma, particularly when ocular treatment is likely to cause severe ocular morbidity (e.g., if the tumour extends close to optic disc or macula). These studies would indicate whether ocular treatment prolongs life in the case of metastasising melanoma (i.e., once metastatic spread has already commenced), pre-metastasising melanoma or both. In practice, there are many obstacles to such trials. First, there are ethical concerns about the dangers of delaying treatment and missing any opportunities for preventing metastasis. Second, it will be difficult to recruit sufficient numbers of patients, because of the rarity of uveal melanomas, particularly of asymptomatic tumours. Third, much data will be lost because treatment becomes necessary for ocular reasons soon after the patient is enrolled (e.g., if the tumour grows or becomes symptomatic). Fourth, even if sequential biopsy is achieved, it will be difficult to determine whether any inconsistent results indicate tumour progression or intra-tumoral heterogeneity.

For these reasons, randomised trials may not be conducted for many years, if at all. In the meantime, multi-centre, prospective cohort studies with matched, treated and untreated patients would seem preferable to performing no investigation at all. If the tumour histology and genetic studies were to be determined sequentially by repeated biopsies, then such studies may reveal which tumours progress without treatment and which do not. Prospective cohort studies should also determine how many untreated patients eventually require some form of intervention as well as providing insights regarding the reasons for such therapy. Importantly, there studies would how whether procrastination has any adverse consequences in terms of mortality, visual loss, ocular conservation, psychological morbidity and cost.

### Routine clinical practice

It is conventional procedure to obtain signed consent for surgery but not for withholding treatment; however, in view of the controversial nature of non-treatment, it would be prudent to obtain written consent from patients whose treatment is deferred. This should prevent subsequent recriminations in the event of metastatic death or other untoward events.

Intuitively, patients tend to believe that surgical treatment of cancer improves survival, but if there is no good scientific evidence for such benefit then this should be explained to them; otherwise, consent for treatment cannot be regarded as truly informed. It would be wise to document that this communication has taken place, especially if iatrogenic ocular morbidity is likely to occur.

As for the management itself, there seem to be few guidelines on how to decide whether to administer treatment when the required evidence is lacking. It is likely that there is much variation in clinical practice. The author attempts to help patients select the management that best fits their wishes and attitude to risk. This can be difficult, especially when there is equipoise between observation, phototherapy, radiotherapy, endoresection and enucleation, as is often the case with small, juxtapapillary melanocytic tumours of indeterminate malignancy. A CD-ROM of the actual conversation is given to the patient to help remember what was said. Some patients express bemusement that they travel a long distance to seek the advice of an ‘expert’ only to be told that they must make up their own minds on what to do about their tumour.

## Conclusions

Formidable logistical and ethical obstacles have prevented randomised controlled trials of treatment *vs* non-treatment of uveal melanoma. It has therefore been necessary to rely on case series, whose results have given rise to a bewildering variety of hypotheses. Because of the lack of good evidence, practitioners and patients rely on intuition. For example, some opinion leaders consider endoresection of choroidal melanoma to be dangerous if not preceded by radiotherapy (Bechrakis *et al*, 2004). This is perhaps because they regard metastatic spread of this cancer to be a mechanistic process, with surgical trauma disseminating malignant cells. Such allegorical thought probably formed the basis of Halsted's mutilating radical mastectomy, with breast cancer being compared to a weed with long roots, all of which had to be dug out entirely if any success was to be achieved.

Uncertainty regarding the impact of ocular treatment on survival has ethical implications, particularly about informed consent for treatment.

There is still much to learn about how best to cope with uncertainty about therapeutic benefit.

## References

[bib1] Augsburger JJ, Correa ZM, Shaikh AH (2009) Effectiveness of treatments for metastatic uveal melanoma. Am J Ophthalmol 148: 119–127 19375060 10.1016/j.ajo.2009.01.023

[bib2] Augsburger JJ, Correa ZM, Trichopoulos N (2007) An alternative hypothesis for observed mortality rates due to metastasis after treatment of choroidal melanomas of different sizes. Trans Am Ophthalmol Soc 105: 54–59; discussion 59–60 18427594 PMC2258111

[bib3] Bechrakis NE, Höcht S, Martus P, Kreusel KM, Heese J, Foerster MH (2004) Endoresection following proton beam irradiation of large uveal melanomas. Ophthalmologe 101: 370–376 15067418 10.1007/s00347-003-0911-2

[bib4] Bove R, Char DH (2004) Nondiagnosed uveal melanomas. Ophthalmology 111: 554–557 15019335 10.1016/j.ophtha.2003.07.002

[bib5] Collaborative Ocular Melanoma Study Group (2006) The COMS randomized trial of iodine 125 brachytherapy for choroidal melanoma: V. Twelve-year mortality rates and prognostic factors: COMS report no. 28. Arch Ophthalmol 124: 1684–1693 17159027 10.1001/archopht.124.12.1684

[bib6] Damato B (2001) Detection of uveal melanoma by optometrists in the United Kingdom. Ophthalmic Physiol Opt 21: 268–271 11430620 10.1046/j.1475-1313.2001.00595.x

[bib7] Damato B (2006) Treatment of primary intraocular melanoma. Expert Rev Anticancer Ther 6: 493–506 16613538 10.1586/14737140.6.4.493

[bib8] Damato B, Coupland SE (2009) A reappraisal of the significance of largest basal diameter of posterior uveal melanoma. Eye 23: 2152–2160 19876071 10.1038/eye.2009.235

[bib9] Damato B, Dopierala J, Klaasen A, van Dijk MC, Sibbring JS, Coupland SE (2009) Multiplex ligation-dependent probe amplification of uveal melanoma: correlation with metastatic death. Invest Ophthalmol Vis Sci 50(7): 3048–3055 19182252 10.1167/iovs.08-3165

[bib10] Damato B, Duke C, Coupland SE, Hiscott P, Smith PA, Campbell I, Douglas A, Howard P (2007) Cytogenetics of uveal melanoma: a 7-year clinical experience. Ophthalmology 114: 1925–1931 17719643 10.1016/j.ophtha.2007.06.012

[bib11] Damato B, Eleuteri A, Fisher AC, Coupland SE, Taktak AF (2008) Artificial neural networks estimating survival probability after treatment of choroidal melanoma. Ophthalmology 115: 1598–1607 18342942 10.1016/j.ophtha.2008.01.032

[bib12] Damato B, Lecuona K (2004) Conservation of eyes with choroidal melanoma by a multimodality approach to treatment: an audit of 1632 patients. Ophthalmology 111: 977–983 15121377 10.1016/j.ophtha.2003.09.028

[bib13] Desjardins L, Dorval T, Lévy C, Cojean I, Schlienger P, Salmon RJ, Validire P, Asselain B (1998) Randomised study on adjuvant therapy by DTIC in choroidal melanoma. Ophtalmologie 12: 168–173

[bib14] Dopierala J, Damato BE, Lake SL, Taktak AF, Coupland SE (2010) Genetic heterogeneity in uveal melanoma assessed by multiplex ligation-dependent probe amplification. Invest Ophthalmol Vis Sci e-pub ahead of print, 19 may 2010; doi:10.1167/10rs.09-5004 10.1167/iovs.09-5004 20484589

[bib15] Ehlers JP, Worley L, Onken MD, Harbour JW (2008) Integrative genomic analysis of aneuploidy in uveal melanoma. Clin Cancer Res 14: 115–122 18172260 10.1158/1078-0432.CCR-07-1825

[bib16] Eskelin S, Kivelä T (2002) Mode of presentation and time to treatment of uveal melanoma in Finland. Br J Ophthalmol 86: 333–338 11864894 10.1136/bjo.86.3.333 PMC1771030

[bib17] Eskelin S, Pyrhönen S, Hahka-Kemppinen M, Tuomaala S, Kivelä T (2003) A prognostic model and staging for metastatic uveal melanoma. Cancer 97: 465–475 12518371 10.1002/cncr.11113

[bib18] Eskelin S, Pyrhönen S, Summanen P, Hahka-Kemppinen M, Kivelä T (2000) Tumor doubling times in metastatic malignant melanoma of the uvea: tumor progression before and after treatment. Ophthalmology 107: 1443–1449 10919885 10.1016/s0161-6420(00)00182-2

[bib19] Gragoudas ES, Lane AM, Munzenrider J, Egan KM, Li W (2002) Long-term risk of local failure after proton therapy for choroidal/ciliary body melanoma. Trans Am Ophthalmol Soc 100: 43–48; discussion 48–9 12545676 PMC1358945

[bib20] Hawkins BS (2004) The Collaborative Ocular Melanoma Study (COMS) randomized trial of pre-enucleation radiation of large choroidal melanoma: IV. Ten-year mortality findings and prognostic factors. COMS report number 24. Am J Ophthalmol 138: 936–951 15629284 10.1016/j.ajo.2004.07.006

[bib21] Kujala E, Makitie T, Kivela T (2003) Very long-term prognosis of patients with malignant uveal melanoma. Invest Ophthalmol Vis Sci 44: 4651–4659 14578381 10.1167/iovs.03-0538

[bib22] Manschot WA, van Strik R (1987) Is irradiation a justifiable treatment of choroidal melanoma? Analysis of published results. Br J Ophthalmol 71: 348–352 3580350 10.1136/bjo.71.5.348 PMC1041164

[bib23] Onken MD, Worley LA, Ehlers JP, Harbour JW (2004) Gene expression profiling in uveal melanoma reveals two molecular classes and predicts metastatic death. Cancer Res 64: 7205–7209 15492234 10.1158/0008-5472.CAN-04-1750 PMC5407684

[bib24] Parrella P, Sidransky D, Merbs SL (1999) Allelotype of posterior uveal melanoma: implications for a bifurcated tumor progression pathway. Cancer Res 59: 3032–3037 10397238

[bib25] Prescher G, Bornfeld N, Hirche H, Horsthemke B, Jöckel KH, Becher R (1996) Prognostic implications of monosomy 3 in uveal melanoma. Lancet 347: 1222–1225 8622452 10.1016/s0140-6736(96)90736-9

[bib26] Straatsma BR, Diener-West M, Caldwell R, Engstrom RE (2003) Mortality after deferral of treatment or no treatment for choroidal melanoma. Am J Ophthalmol 136: 47–54 12834669 10.1016/s0002-9394(02)02270-5

[bib27] Tschentscher F, Hüsing J, Hölter T, Kruse E, Dresen IG, Jöckel KH, Anastassiou G, Schilling H, Bornfeld N, Horsthemke B, Lohmann DR, Zeschnigk M (2003) Tumor classification based on gene expression profiling shows that uveal melanomas with and without monosomy 3 represent two distinct entities. Cancer Res 63: 2578–2584 12750282

[bib28] Voelter V, Schalenbourg A, Pampallona S, Peters S, Halkic N, Denys A, Goitein G, Zografos L, Leyvraz S (2008) Adjuvant intra-arterial hepatic fotemustine for high-risk uveal melanoma patients. Melanoma Res 18: 220–224 18477897 10.1097/CMR.0b013e32830317de

[bib29] White VA, Chambers JD, Courtright PD, Chang WY, Horsman DE (1998) Correlation of cytogenetic abnormalities with the outcome of patients with uveal melanoma. Cancer 83: 354–359 9669819

[bib30] Zimmerman LE, McLean IW, Foster WD (1978) Does enucleation of the eye containing a malignant melanoma prevent or accelerate the dissemination of tumour cells. Br J Ophthalmol 62: 420–425 352389 10.1136/bjo.62.6.420 PMC1043246

